# A single nucleotide polymorphism in Wilms’ tumor 1 gene and the risk of acute myeloid leukemia in Sudan

**DOI:** 10.1186/1471-2164-15-S2-P43

**Published:** 2014-04-02

**Authors:** Amr A Ahmed, H B Eltahir, Malik HI Mustafa, Munsoor M Munsoor

**Affiliations:** 1 Alnomais Medical & Pharmacies Group, Abha, Saudi Arabia; 2 Department of Biochemistry, Faculty of Medicine, University of El Imam El Mahdi, Sudan; 3 Department of Medical Lab Sciences, College of Applied Medical Science, Taif University, Saudi Arabia; 4 Department of Heamatology, Faculty of Medical Laboratory Science, Sudan University of Science and Technology, Sudan

## Background

The diagnosis, prognosis, and treatment of acute myeloid leukemia (AML) have been transformed to be based on genetic, genomic, and molecular characteristics of the disease. Wilms' tumour (WT1) is an important regulatory molecule involved in cell growth and development [1-3]. WT1 is highly expressed in the bone marrow or peripheral blood of a variety of leukemia.

## Materials and methods

This study was designed to investigate the involvement of WT1 gene mutation in the development of Acute Myeloid Leukemia in Sudan. The study involved 51 patients and 75 healthy controls. Genomic DNA was isolated from peripheral blood leukocytes using salting out method. PCR amplification of the target sequence (214 bp) within exon7 of WT1 gene was carried out. The known A→G transition mutation that destroys an AflIII restriction enzyme recognition site was detected by RFLP analysis.

## Results

The study revealed that 43.14% of cases (22/51 patients) were heterozygous A/G and only 3.92% (2/51) were homozygous G/G for the mutant allele on the other hand 26.67% (20/75 controls) were heterozygous A/G and the homozygous G/G genotype was not observed in any of the controls. The study showed a significant difference in the frequency of the mutant WT1 allele between patients 25.49% and controls 13.33%.

## Conclusions

In the Sudanese population, the Wilms' tumour (WT1) gene A→G mutation appears to be associated with increased risk of developing Acute Myeloid Leukemia. The disease was found to be associated with the heterozygous genotype.
